# The impact of air pollution on the incidence and mortality of COVID-19

**DOI:** 10.1186/s41256-020-00167-y

**Published:** 2020-09-01

**Authors:** Abhinav Karan, Kabeer Ali, Surujpal Teelucksingh, Sateesh Sakhamuri

**Affiliations:** Department of Clinical Medical Sciences, Faculty of Medical Sciences, The University of the West Indies, St. Augustine Campus, St. Augustine, Trinidad and Tobago

**Keywords:** COVID-19, SARS-CoV 2, Air pollution, Particulate matter, Household air pollution

## Abstract

Air pollution is the most significant environmental risk factor for all-cause mortality, and it has caused substantial disability-adjusted life-years and economic loss. Air pollution intensified the mortality during past pandemics, Spanish flu in 1918 and SARS-CoV-1 in 2003. It increases host susceptibility and virulence of respiratory infections and reduces viral clearance. Thus, a question arises whether there will be any impact of air pollution on the current pandemic coronavirus disease 2019 (COVID-19)? Thus far, history and science are directing towards an immense potential impact of air pollution on the COVID-19 pandemic. Some of the devastated countries with the current pandemic are those with a poor air quality index. Further epidemiological and ecological studies are necessary to confirm this association. Also, countries must mobilize funding for mitigation of air pollution to benefit environmental health and ameliorate its potential effects on pandemics of the future.

## Background

The world is currently gripped in a planetary health crisis, expected to cause at least 4.2 million deaths this year; that of atmospheric pollution. Polluted air is the most significant environmental risk factor for all-cause mortality. It has increased the risk of cancer, chronic pulmonary and cardiovascular diseases, and caused the loss of at least 100 million disability-adjusted life-years (DALYs) and US$225 billion annually [1]. In the midst of our unprecedented coronavirus pandemic, the morbidity posed by air pollution and its impact on our current situation must not be forgotten. Mortality from air pollution, especially during pandemics, cannot be understated. The devastating 1918 Spanish Influenza Pandemic saw a 10% increase in mortality in large coal-capacity cities. During the first pandemic of the current century, severe acute respiratory syndrome-associated coronavirus-1 (SARS-CoV-1) in 2003, patients from areas with high air pollution indices (API) displayed a 200% increased relative risk of death compared to people from areas with a low API [2]. Despite this, 91% of the world’s population lives in areas exceeding the recommended World Health Organization (WHO) air quality limits. Thus, a question arises whether there will be any impact of air pollution on the current pandemic coronavirus disease 2019 (COVID-19)?

## Effect of air pollutants on host susceptibility to infection

Air pollutants increase host susceptibility to respiratory viral infections by increasing epithelial permeability to viral receptors and reducing surfactant production [3]. They also reduce viral clearance by impairing macrophage-mediated phagocytosis and antigen presentation, expression of both the natural killer and cytotoxic T cells, and allowing viral proliferation, thereby negatively influencing the ability of the host to respond appropriately to infection. Furthermore, air pollutants increase the virulence of respiratory infections. Nitrogen dioxide (NO_2_) decreases the minimal infectious dose of murine cytomegalovirus by a factor of 100 and increases rhinovirus infectivity by upregulating its viral receptor. In Italy, a study showed an increase in the environmental concentration of NO_2_ correlated with an increase in acute respiratory infections by 4% [3]. Exposure to Sulphur dioxide (SO_2_) has also been associated with an increase in influenza infections [3]. Particulate matter, mainly a diameter of less than 2.5 μm (PM_2.5_), can reduce phagocytosis of viruses, promote their proliferation, and produce a significant proinflammatory state by inducing the release of cytokines IL-1, IL-6, and TNF-α from alveolar macrophages. Resultant inflammation can lead to worsening of pre-existing pulmonary morbidities, asthma and chronic obstructive pulmonary disease (COPD) and increase the risk of cardiac disease by altering cardiac autonomic function and accelerating atherosclerosis. Furthermore, acute and sub-chronic exposure to particulate matter upregulates the angiotensin-converting enzyme 2 (ACE2) receptor, which plays a critical role in the invasion of the SARS-CoV-2 virus into the respiratory epithelium [4]. A statistically significant link has also been demonstrated between acute respiratory distress syndrome (ARDS), and air pollution in the form of PM_2.5_ and ozone, particularly in elderly adults. This is relevant to COVID-19, given the high rate of death from ARDS [5].

## Predisposition due to household air pollution

Often overlooked is the impact of household air pollution (HAP) on susceptibility to respiratory infections. HAP accounts for nearly 16% of the global disease burden from ambient air pollution, accounting for nearly 3.55 million deaths in 2010, and consequently, its impact must not be ignored. A third of the world uses solid fuel for cooking and heating coupled with concomitant cigarette smoking, which can cause epithelial inflammation and predispose acute lower respiratory infections. HAP can cause dysregulation of the antioxidant: oxidant ratio, reducing antioxidant concentration and promoting oxidative stress. HAP also changes the defense mechanism against infection in the lung, and it is highly plausible that this modification of natural lung flora can result in an increased risk of pulmonary infections. As an example, cigarette smoke can increase the receptor-dependent adhesion of Streptococcus sp. to the respiratory epithelium. The magnitude of exposure to HAP is also inextricably linked to poor socioeconomic status and noteworthy that the global distribution of poverty and HAP are almost identical. It can be postulated that this exposure, particularly in developing countries or the urban poor in rich countries across the globe, places individuals at high risk of more severe outbreaks of COVID-19 [6].

## Early evidence on COVID-19

Thus far, history and science are directing towards an immense potential impact of air pollution on the COVID-19 pandemic. Some of the most devastated countries are those with a poor air quality index, such as China, Northern Italy, and New York City. Considering Northern Italy, in particular, Lombardy and Emilia Romagna are among the most polluted areas in Europe. Mortality here was 12% compared to the rate of 4.5% elsewhere across Italy [7]. In fact, across more than 3000 counties of the United States, an increase of 1 μg/m^3^ in PM_(2.5)_ has been shown to increase mortality from COVID-19 by 8%, and in New York state alone, by 15% [5]. Like pandemics before, there is likely a positive correlation between air pollutants and the incidence and mortality from COVID-19, but further epidemiological studies are necessary to confirm this hypothesis.

A study recently assessed tropospheric NO_2_ levels, a marker of atmospheric pollution, in four European countries, Italy, France, Spain and Germany. Chronic NO_2_ exposure has been linked to background diseases such as hypertension and responsible for the synthesis of proinflammatory cytokines, which are linked to increased COVID-19 mortality. Of five identified fatality hotspots in Italy and Spain, which accounted for 78% of all deaths, all were shown to have concentrated air pollution [8]. Additionally, in England, both the highest incidence and case-fatality rates occurred around London and the Midlands- regions with the highest concentration of air pollutants. The inverse was seen in regions with less air pollution, giving further support to this notion [9].

In fact, SARS-CoV-2 RNA has been isolated from particulate matter in a study conducted in Bergamo, Northern Italy, suggesting that particulate matter in air pollution may even act as a vector for transmission of COVID-19. This may serve as a possible explanation for a higher COVID-19 burden in areas with high air pollution, and may also be used as an indicator for epidemic recurrence [10].

## Imperative interventions

The evidence presented above should serve as a wake-up call for decision-makers keen to advocate planetary health. It is our opinion that there is an unsatisfactory response by many environmental agencies to regulate air pollution, particularly based on the mounting evidence of the influence of air pollution on pandemics of past, present and almost certainly future.

The Paris Agreement, which calls for limiting the influence of climate change, emphasizes the importance of reducing air pollution as a crucial component behind its success. It is crucial that countries globally, now more than ever, appreciate the association between climate change and air pollution, and reduce air pollutants through aggressive policy interventions, not only to potentially reduce the impact of COVID-19 and improve human health, but also to limit climate change.

## Conclusion

The link between air pollution and morbidity and mortality brings a sobering revelation in the wake of the tremendous economic and social upheaval caused by SARS-CoV-2. There must be a continued affirmation of the commitment to the global fight against climate change by meeting the Paris Agreement's goals, which can save up to 1 million lives a year from mitigation of air pollution alone. Countries must mobilize funding for air pollution mitigation, establish new systems for pollution monitoring such as satellite imaging, and build multi-sectoral partnerships for pollution control, to benefit environmental health and ameliorate its potential effects on the current and future pandemics.

## Data Availability

Not applicable.

## Abbreviations

ACE-2: Angiotensin converting enzyme-2
API: Air pollution index
COPD: Chronic obstructive pulmonary disease
COVID-19: Coronavirus disease 2019
DALYs: Disability-adjusted life-years
HAP: Household air pollution
IL: Interleukin
NO_2_: Nitrogen dioxide
PM: Particulate matter
SARS-CoV: Severe acute respiratory syndrome-coronavirus
SO_2_: Sulphur dioxide
TNF: Tumour necrosis factor
USA: United States of America
WHO: World health organization
